# Promotion strategy for online healthcare platform during the COVID-19 pandemic: Evidence from Spring Rain Doctor in China

**DOI:** 10.3389/fpsyg.2022.960752

**Published:** 2022-11-30

**Authors:** Lanting Zhang, Dan Lv, Weijia Li, Zeyu Xing

**Affiliations:** ^1^School of Economics and Management, Harbin Engineering University, Harbin, China; ^2^School of Management, Zhejiang University of Technology, Hangzhou, China

**Keywords:** online healthcare platform, evolutionary game theory, tripartite stakeholders, COVID-19, China

## Abstract

**Introduction:**

Online healthcare platform (OHP) is a new form of medical treatment that solves the problems of an unbalanced distribution of medical resources in China. Especially during the COVID-19 pandemic, OHP has greatly reduced the medical pressure of the hospital and the risk of cross-infection.

**Methods:**

Based on self-determination theory (SDT) (Ryan and Deci, 2000), privacy calculus theory (PCT) (Culnan, 1999) and perceived value theory (PVT) (Choi, 2004), this study uses evolutionary game theory to analyze behavioral strategies and their dynamic evolution in the promotion of OHP. Moreover, we conduct numerical simulations with the help of program compilation.

**Results:**

The results demonstrate that (1) both the qualification inspection of doctors and the investment in information protection influence doctors’ participation in and patients’ usage of OHP; (2) both the initial probabilities of doctor participation and patient usage influence the multi-game results; (3) the trend of doctors joining OHP is affected by registration cost, time cost, and reputation loss; and (4) the trend of patients using online healthcare is mainly decided by the cost.

**Conclusion:**

This study takes the Spring Rain Doctor as an example to verify the game results. To further popularize online medical treatment among patients, the platform should attach importance to the inspection of doctors and the protection of privacy information and strengthen its publicity in remote places.

## Introduction

In the wake of the COVID-19 outbreak, the international environment has become more complex and uncertain. The new scientific and technological revolution and industrial transformation continue to evolve, and new challenges and opportunities keep emerging. Digital technology and the digital economy are the forerunners of the world scientific and technological revolution and industrial transformation. Online healthcare is an important way in which the digital economy works during the COVID-19 pandemic. The online healthcare platform (OHP) is an Internet platform wherein doctors provide healthcare services and scientific knowledge to patients directly without time and space restrictions. Patients seek targeted treatment solutions through online medical consultation services (Ye et al., 2022). In 2020, the outbreak of novel coronavirus pneumonia (COVID-19) has seriously affected people’s health and normal life (Wang et al., 2021). Due to the outbreak of the COVID-19, the public was mostly anxious about going to the hospital, especially for patients with mild cold symptoms. Therefore, the OHP has received unprecedented attention due to its benefits such as convenience, cost, flexibility, and time savings (Whitten et al., 2007; Jung and Padman, 2015). Compared with traditional offline health care, the OHP can prevent COVID-19 infections, as patients can communicate with doctors and obtain health information anytime and anywhere (Ren and Ma, 2020). In the process of prevention and control of the epidemic, the Chinese OHP has played the unique advantages of online consultation. OHP has reduced the risk of patients’ infection and alleviated the situation of medical resources shortage in some areas (Wang et al., 2021). Simultaneously, the COVID-19 epidemic catalyzed the expansion of OHP, represented a major opportunity for internet-based medical treatment in the medium and long term. According to China Internet Network Information Center’s 49th statistical report on the Development of Internet in China, by the end of 2021, the scale of online medical care in China had reached 298 million, with a year-on-year growth of 24.2%. It shows that OHPs are a valuable resource in dealing with large-scale public health emergencies (Jiang et al., 2021).

In China, the Chinese government had adopted a series of administrative measures to encourage the development of OHP, especially during the COVID-19 pandemic. The State Council, the National Health Commission and other departments have issued a series of policies related to Internet healthcare, including the “Opinions on Promoting the Development of Internet + Medical and Health,” aiming to build and standardize the domestic Internet healthcare system. The online healthcare industry continued to maintain rapid development, and more Internet companies joined the competition. As of December 2021, the utilization rate of online healthcare in China was 28.9%, an increase of 7.2 percentage points compared with December 2020 (China, Internet Network Information Center, 2022). The promotion of OHP can bring many benefits to patients and doctors. For patients, OHP can reduce transaction costs brought by geographical and time constraints, while improving convenience (Deng and Liu, 2017; Wu and Lu, 2018). OHP can support patient self-health management by promoting the sharing of health-related knowledge with patients (Yang et al., 2019b). By browsing the doctor’s homepage on the platform, patients can view the doctor’s information and understand his/her training, professionalism, and treatment effectiveness (Cao et al., 2017). After the outbreak of COVID-19, online health services satisfied peoples’ needs for convenient and contactless health services (Jiang et al., 2021). In addition, according to self-determination theory (SDT; Ryan and Deci, 2000), doctors take the behavior of participating in OHP because they can obtain the relevant value to satisfy their inner needs. Doctors can improve the usage of their time by answering patients’ questions in their spare time through OHP (Lu et al., 2019). They can also actively use OHP to obtain economic and social benefits (Jiang et al., 2021; Zhang et al., 2021). Simultaneously, OHP can promote doctor–patient communication to a certain extent, reduce the pressure of hospital diagnosis and treatment, and resolve medical resource shortages and doctor–patient disputes (Luo et al., 2019; Li C. R. et al., 2020). Patients in remote and severely affected areas can receive remote diagnosis and treatment, so as to realize information sharing and diagnosis and treatment across regions and departments (Liang et al., 2020). Therefore, the promotion of OHP is of great significance for the prevention and control of the COVID-19 pandemic.

However, the rapid development of online healthcare has also brought many problems caused by the imperfection of relevant laws and regulations for OHP in China, such as the lack of effective supervision of platform, uneven quality of online medical platforms, low barriers to entry, and the low degree of specialization (Zhang and Feng, 2019). Some platforms have underinvested in information maintenance, leading patients to face the risk of private information leakage (Zhu et al., 2018a). Leaks of disease-related information can lead to social stigma, disease discrimination, mental stress, and a series of other issues (Backerman, 2008). Privacy calculus theory (PCT) points out that when users disclose personal information in exchange for some economic or social benefits, they will make an evaluation to ensure that their private information will not be used illegally and they will not be negatively affected (Culnan and Armstrong, 1999). Therefore, patients’ concern about privacy risks is an important factor affecting their use of OHP. Moreover, the OHP lacks strict inspection of doctors’ qualifications and trainings, and the quality of doctors’ medical services varies, leading to misdiagnosis and reduction in patient satisfaction (Yang et al., 2019a). Meanwhile, this will damage the reputation of doctors. According to perceived value theory (PVT), perceived value refers to the comparison of benefits and costs in the process of using a product or service. Perceived values are considered to have positive effects on actual users’ attitudes and long-term usage behavior (Hu, 2021). Perceived value can be measured by perceived benefits and perceived costs (Dong, 2019). Patients and doctors will use and participate in OHP when the perceived benefits outweigh the perceived costs. Therefore, we need to explore the influencing factors of doctor participation and patient use from the perspective of benefits and costs.

Based on SDT, PCT and PVT, we collated the factors that influence patient use and doctor participation. But the spread of OHP among patients and doctors is a long-term process. In this process, the behaviors of patients, doctors and platforms will interact with each other. Information asymmetry exists among participants, and the behavior of subjects is characterized by bounded rationality. Therefore, there is a game between the three participants. The evolutionary game model is based on the assumption of incomplete rationality of human beings, which can better reflect the complexity and uncertainty of the subject’s behavior, and describe the detailed process of the subject’s decision-making eventually reaching dynamic equilibrium over time (Weibull, 1995). Therefore, this article integrated evolutionary game theory, SDT, PCT and PVT to construct a tripartite evolutionary game model of doctor, patient and platform. We used numerical simulation to verify the accuracy of the model and evolution results. Finally, we used the case of Spring Rain doctor platform to verify the simulation results, and put forward management suggestions for the promotion of OHP and the improvement of Internet medical treatment.

## Literature review

Scholars usually study OHP from the perspectives of patients, doctors, and platforms. We can also determine the factors affecting the promotion of OHP from these three aspects.

Existing literature has mainly focused on the influence factors of patients’ behavior from different theoretical perspectives. Based on PVT, Choi et al. (2004) showed that in the choice of online medical services, customer perceived value has a significant impact on their satisfaction and behavioral intentions. Deng et al. (2014) determined that perceived value, attitude, perceived behavior control, technology anxiety, and self-actualization need positively affect the behavior intention of older users. Gu et al. (2018) founded a positive effect of perceived usefulness on patient satisfaction with OHP. Moreover, the performance expectations, social influence, and credibility of network health information are the key factors affecting patients’ acceptance and use of OHP (Sun and Lu, 2014). Yang et al. (2019c) proposed that the response time, interaction depth and service content of the first consultation affected the patient’s perceived trustworthiness of the doctor, which had a significant impact on the patient’s follow-up consultation behavior. Simultaneously, service prices also affect satisfaction, and they show an inverted U-shaped relationship (Wu and Lu, 2018). Shao et al. (2022) examined the impacts of different incentive levels including identity incentive, privilege incentive, and material incentive on user perceived value, user engagement, and user loyalty. Based on PCT. Wang et al. (2017) founded that privacy calculation has an important impact on the willingness to use OHP. Individuals will weigh the disclosure of privacy information and the acquisition of benefits, and decide whether to use OHP. Sun et al. (2022) supposed that patients tend to feel insecure when using the OHP to participate in various activities. They believed that security control of OHPs negatively influences privacy concerns and patients’ trust in platform, and further influences patients’ satisfaction and willness to use. Aligned with the unified theory of acceptance and use of technology (UTAUT), Nunes et al. (2019) examined the moderating roles of age, gender, and smartphone experience in the relationship between technology acceptance determinants (performance expectancy, effort expectancy, social influence, and facilitating conditions) and the intention to use mobile health applications.

Most studies have been conducted from the perspective of patients. Although understanding how patients benefit from OHP is imperative, OHP will be viable only when participating doctors also gain returns from OHP (Guo et al., 2017). OHP requires significant engagement from doctors, as they provide doctor-driven services (Liu Q. B. et al., 2020). In OHP, doctors provide consultation services, knowledge, and information to help patients understand their diseases and obtain treatments (Li et al., 2019). Doctors’ contributions, such as providing consultation services and healthcare information, promote the development of such platforms in the long term (Yang et al., 2019c). Therefore, some scholars have paid attention to the influencing factors of doctors joining OHP based on different theoretical perspectives. For instance, Guo et al. (2017) used social exchange theory to argue thatdoctor participation in OHCs is a social exchange behavior, and studied the impact of status capital and decision capital on the social and economic returns of different doctor groups. The results show that the doctor’s decision capital is a professional component, which is important to the platform maintaining exchange returns. Meanwhile, Chen et al. (2020) combined the expectancy theory and the model of Bagozzi, Dholakia, and Basuroy. The results reveal that extrinsic motivations (i.e., extrinsic rewards, expected relationships, and image) and intrinsic motivation (i.e., a sense of self-worth) significantly influence the desire to serve patients well, which in turn positively affects the willingness to offer free and paid services. Ma and Wang (2018) determined the positive impact of performance expectations, effort expectations, social impact, and convenience conditions on doctors’ willingness to use and usage behavior. Based on SDT, Yang and Zhang (2019) thought that doctors would obtain social support from interaction with patients, and this support could promote doctors’ behaviors in OHP. Meanwhile, Yang et al. (2019c) determined that reputation, monetary rewards, and doctor–patient interaction positively influence doctors’ contribution to OHP, and that the doctor’s professional status moderates the relationship between motivators and the resulting contribution. Moreover, doctors participate in OHP to provide healthcare services to patients and become popular among users (Liu S. et al., 2020). Furthermore, doctors deliver heightened reputation and other returns while improving the provision of healthcare services (Li J. Y. et al., 2020).

From the perspective of platforms, scholars usually study the impact of platforms on society. For instance, Yang et al. (2015) determined that OHP can solve the problem of information asymmetry between doctors and patients. Moreover, in a study on the information transfer between patients and doctors, OHP is found to increase the communication between patients and doctors and improve the quality of care (Mcgeady et al., 2008). Meanwhile, Goh et al. (2016) studied the social value of OHP services. He believed that the OHP can make medical information break through the geographical boundaries and pass from the city to the countryside, which greatly improve the rural medical environment and achieved social value. Moreover, Venkatesh et al. (2016) found that the emergence of Internet medical services could help pregnant women in rural areas obtain online healthcare information. It can alleviate the problem of uneven distribution of medical resources in urban and rural areas and greatly reduce the infant mortality rate. Furthermore, Wu and Zhou (2017) found that the OHP can use the Internet to promote the flow of high-quality doctor resources and medical information services across regions, which is beneficial to improve the uneven distribution of medical resources in China.

To sum up, online healthcare research has mainly focused on the willingness and motivation of patients and doctors. Scholars have studied the behavior and motivation of patients, doctors and other users according to relevant theories of psychology, economics and management. Although some scholars have studied the impact of platforms on society, few studies have focused on the influencing factors of platforms’ behaviors. Meanwhile, there has been little in-depth analysis of the game between doctors, patients and platforms.

There are three innovations in this study compared with other studies. First, although many scholars have studied the influencing factors of patients’ use and doctors’ participation in OHP, due to the information asymmetry among doctors, patients and platforms, the behavioral decisions of all parties show the characteristic of bounded rationality. Some scholars have used evolutionary game theory to study the behavioral evolution of OHP subjects before (Hu, 2020), but few studies have discussed the behavioral interaction and strategy evolution of doctors, patients, and platforms. Therefore, this article introduces evolutionary game theory into the study of OHP promotion strategy, and discusses how to promote OHP from the perspectives of patient use and physician participation.

Secondly, through literature review, we found that most of the factors affecting patients’ willingness to use and satisfaction are related to perceived value and privacy leakage, and most of the factors affecting doctors’ participation in OHP are related to economic benefits, time costs and reputation benefits. Therefore, based on the PVT and PCT, we conducted a study on the influencing factors of patients’ use of OHP. SDT can explain the motivation of individual behavior, so it can be the theoretical basis for influencing factors of doctor participation behavior. This article integrates evolutionary game theory, SDT, PVT and PCT, uses evolutionary game model and numerical simulation method to explore the law of behavioral evolution of the three participants. At the same time, factors such as economic benefits, privacy leakage, perceived value, reputational risk are incorporated into the game model.

Thirdly, based on the use of evolutionary game and numerical simulation, this article verifies the numerical simulation results through case analysis of Spring Rain Doctor platform. Spring Rain Doctor is the first Internet enterprise in China to try online healthcare. We believe that Spring Rain Doctor is representative among Chinese OHPs. The case analysis makes the conclusions and suggestions more practical and more in line with the specific situation of China. This work is of great importance to improving the imbalance of medical resources during the COVID-19 pandemic.

## Materials and methods

### Evolutionary game theory in healthcare

Evolutionary game theory is derived from the concept of evolutionary stability strategy (Smith, 1974). It was originally developed in the economics field to study social interactions (Li, 2011). This method takes the group of participants with limited rationality as the research object and examines the evolution trend of group behavior from the viewpoint of system theory. Over the last few decades, the evolutionary game theory has been widely adopted by economists, sociologists, social scientists, and the philosophers (Xia et al., 2017).

The concept of evolutionary game theory has also been used to study the actions of players in healthcare systems. Chen et al. (2018) leveraged the evolutionary game theory to build a novel model to capture the behaviors of hospitals and patients in mHealth. Then, they analyzed the payoff matrix between hospitals and patients such that a replicator dynamic system can be built. Moreover, Yu et al. (2020) used evolutionary game theory to analyze behavioral strategies and their dynamic evolution in the implementation and operation of telemedicine. From the perspective of privacy disclosure, Xu et al. (2022) constructed an evolutionary game model of privacy disclosure behavior with users and online health communities as the main participants. Zheng and Xu (2021) discussed the complex institutional environment faced by OHP, believed that there was a dynamic game among the government, platform and doctors, and used the evolutionary game method to build a game model of the government, doctors and platform. Taking the mHealth system as the context, Zhu et al. (2018b) builded an evolutionary game to model three types of entities (including system providers, hospitals and governments) under the conditions of incomplete information and bounded rationality.

However, no research has used evolutionary game method to model doctors, patients and platforms. Each of the three participants had a choice of two strategies. Firstly, doctors can choose to participate in OHP, but doctors also pay time costs and registration costs, and may also lose reputation. Secondly, patients can choose to use OHP, but when patients face the risk of privacy leakage and misdiagnosis, patients can also not use OHP and look for alternative solutions. Thirdly, platforms can choose to increase qualification review and privacy protection, but at the same time, platforms need to pay economic costs. There may be situations where the platform sells or discloses users’ private information to third parties for the benefit of third parties. Meanwhile, the strategic choices of the three participants will have an impact on each other’s strategic choices. Therefore, we believe that the evolutionary game method is suitable for this study. By building an evolutionary game model, we can fully consider the benefits and costs of the three participants of OHP, so as to explore the action strategies of the three participants, and finally put forward suggestions for the application and promotion of OHP.

### Evolutionary stable strategy

Smith (1976) devised a central concept of evolutionary game theory called the evolutionary stable strategy (ESS). ESS is the strategy when game players continuously learn and imitate successful strategies in the evolution process and finally reach a stable state after improving their own strategies. The replication dynamic equation is a dynamic differential equation, which is used to express the frequency that a particular strategy is selected by a class of groups. It can be expressed by the following equation:


$$\frac{dx_{i}}{dt}=x_{i}\left(u_{si}-\bar{u}\right)$$

where *x*_i_ denotes the frequency of strategy *s*_i_, *u*_si_ denotes the expected return of strategy *s*_i_ selected by this group, and $\bar{u}$ denotes the average expected return of this group. When interference factors make *x* smaller than x^*^, dx/dt needs to be bigger than 0. Moreover, when *x* is bigger than x^*^, dx/dt needs to be smaller than 0 to achieve a stable state.

In summary, evolutionary game theory is based on the assumption of bounded rationality, considering the interaction between game players. Through multiple games, players constantly learn and improve their strategies and finally reach an evolutionary stable state.

## Tripartite evolutionary game model of stakeholders in OHP

### The hypothesis of the tripartite evolutionary game model

In China, the enterprise-based mode is the main mode of online healthcare, accounting for 70% of the total. Internet companies build OHP and cooperate with doctors. The platforms need to contact doctors and inspect the qualifications of doctors. During this time, the platforms should pay more costs. In addition, the platform must invest in information protection. The higher platforms invest, the higher the risk of cost recovery that platforms confront.

From the perspective of doctors, doctors can obtain economic and social benefits by joining OHP (Guo et al., 2017). Specifically, doctors can obtain additional income and build a reputation by providing good online healthcare services (Chang et al., 2019; Chen et al., 2020). In OHP, reputational reward obtained by doctors mainly comes from the patients’ feedback such as their ratings and reviews (Liu et al., 2016). The reputation reward is especially crucial for doctors to enhance their own career and occupational influence (Liu et al., 2016; Yang and Zhang, 2019). Simultaneously, doctors must pay the time and registration costs. When the platforms do not strictly inspect or evaluate doctors, doctors will face reputation loss due to misdiagnosis and low patient satisfaction. Moreover, when the platforms’ investment in information protection is insufficient, doctors will face the risk of leakage of private information.

From the patients’ perspective, patients can save time and money by using OHP. However, patients may face the risk of being misdiagnosed and privacy.

The following hypotheses were tested in this study:

1. Doctors take two courses of action: One strategy is to join OHP (joining), and the other is not to join OHP (not joining). Thus, the strategy space of doctors is S1 (joining, not joining). Patients take two courses of action: One strategy is to use OHP, and the other is not to use OHP. Thus, the strategy space of patients is S2 (using, not using). OHPs take two courses of action: One strategy is to provide standardized online healthcare services, and the other is not to provide standardized online healthcare services. Thus, the strategy space of platforms is S3 (providing, not providing).
2. The assumptions are that doctors with *x* probability may adopt the “joining” strategy, and those with (1 − *x*) probability may adopt the “not joining” strategy. Patients with *y* probability may adopt the “using” strategy, and those with (1 − *y*) probability may adopt the “not using” strategy. Platforms with *z* probability may adopt the “providing” strategy, and those with (1 − *z*) probability may adopt the “not providing” strategy, in which 0 < *x* < 1, 0 < *y* < 1, and 0 < *z* < 1, respectively.
3. When the doctors join and the patients use OHP, the doctor’s income is represented by *r*_1_, the costs of the doctor’s OHP registration and qualification certification are *c*_1_, and, the doctor’s time costs are *c*_2_. Moreover, *α* and *β* denote the platforms’ qualification inspection strength coefficient (QISC) and information protection investment strength coefficient (IPISC), respectively. When the platforms do not provide standardized online healthcare services, the QISC of doctors is less than 1. It may lead to misdiagnosis or low patient satisfaction. The reputation loss suffered by the doctor is denoted by (1 − *α*)*c*_3_. Simultaneously, the platforms’ IPISC is less than 1, and the information leakage loss suffered by doctors is denoted by (1 − *β*)*c*_4_.
4. The health benefit of patients using OHP is denoted by *e*_1_. When patients choose to go to the hospital instead of using OHP, the health benefit they obtain is *e*_2_, *e*_1_ < *e*_2_. The time and money saved by patients using OHP are represented by *e_3_*. When doctors join OHP, patients use OHP, and platforms provide standard online healthcare services, a solid trust relationship is established among patients, doctors, and platforms. At this time, the patients obtain an additional benefit of *L*. The costs of patients using OHP are *h*_1_. However, when doctors join OHP, patients use OHP, and the platforms do not provide standardized online healthcare services, the health loss of patients is (1 − *α*)*h*_2_, and the loss of patients’ disease information leakage is (1 − *β*)*h*_3_. Regardless of whether doctors join OHP, when patients use OHP and the platforms do not provide standardized online healthcare services, the loss of the patient’s identity information leakage is (1 − *β*)*h*_4_.
5. When the platforms provide standardized online healthcare services, doctors join OHP, and patients use OHP, the economic benefit obtained from the platform is *w*_1_. At this point, the social reputation income obtained by the platforms is *M*. When the platforms provide standardized online healthcare services, the platforms’ QISC for doctors is 1, and the its IPISC is 1. The costs of platforms’ inspection of doctors’ qualification and information protection investment are *t*_1_ and *t*_2_, respectively. When the platforms do not provide standardized online healthcare services, the cost of inspecting doctor qualification is *αt*_1_, and the cost of information protection investment is *βt*_2_, 0 < *α* < 1, 0 < *β* < 1. When the platforms do not provide standardized online healthcare services, the compensation for the patients’ health loss is (1 − *α*)*F*_1_, and the compensation for the patients’ disease information leakage is (1 − *β*)*F*_2_. Meanwhile, the compensation for the leakage of patients’ identity information and the doctors’ information is denoted by (1 − *β*)*F*_3_ and (1 − *β*)*G*, respectively. When patients use OHP, doctors join OHP, and the platforms do not provide standardized online healthcare services, the social reputation loss suffered by the platforms is denoted by *N*.

### Payoff matrix of the tripartite evolutionary game in OHP

Based on the above assumptions, a tripartite evolutionary game model including doctors, patients, and platforms under bounded rationality was constructed. The payoff matrix of the three groups is shown in Table 1.

**Table 1:** Payoff matrix.

	Provide (*z*)		Not provide (1−*z*)	
	Join (*x*)	Not join (1−*x*)	Join (*x*)	Not join (1−*x*)
Use (*y*)	*r*_1_−*c*_1_−*c*_2_	0	*r*_1_−*c*_1_−*c*_2_*−*(1−*α*)*c*_3_−(*1*−*β*)*c*_4_	0
	*e*_1_+*e_3_−h*_1_+*L*	*e*_2_	*e*_1_*+e*_3_*−h*_1_−(1−*α*)*h*_2_*−*(1−*β*)(*h*_3_*+h*_4_)	*e*_2_−(1−*β*)*h*_4_
	*w*_1_−*t*_1_−*t*_2_+*M*	*−t*_1_ *−t*_2_	*w*_1_−*αt*_1_−*βt*_2_−(1*−α*)*F*_1_−(1*−β*)(*F*_2_*+F*_3_*+G*)−*N*	*−αt*_1_*−βt*_2_*−*(1−*β*)*F*_3_
Not use (1−*y*)	*−c*_1_	0	*−c*_1_*−*(1−*β*)*c*_4_	0
	*e_2_*	*e*_2_	*e*_2_	*e*_2_
	*−t*_1_ *−t*_2_	*−t*_1_ *−t*_2_	*−αt*_1_−*βt*_2_−(1−*β*)*G*	−*αt*_1_−*βt*_2_

## Analysis of the tripartite evolutionary game model in OHP

### Replicator dynamics equation of the tripartite evolutionary game

According to the payoff matrix, we separately listed the replication dynamic equations of doctors, patients and platforms.

Under the aforementioned assumption, when doctors implement the “join” strategy, the marginal expected revenue is


$$\begin{array}{c} P_{11}=yz\left(r_{1}-c_{1}-c_{2}\right)+y\left(1-z\right)\\ \left[r_{1}-c_{1}-c_{2}-\left(1-\alpha \right)c_{3}-\left(1-\beta \right)c_{4}\right]+\\ \left(1-y\right)z\left(-c_{1}\right)+\left(1-y\right)\left(1-z\right)\left[-c_{1}-\left(1-\beta \right)c_{4}\right] \end{array}$$

Meanwhile, when doctors implement the “not join” strategy, the marginal expected revenue is


$$P_{12}=yz\left(0\right)+y\left(1-z\right)\left(0\right)+\left(1-y\right)z\left(0\right)+\left(1-y\right)\left(1-z\right)\left(0\right)$$

Moreover, the expected revenue of the doctors is calculated as follows:


$$P_{1}\left(x\right)=xP_{11}+\left(1-x\right)P_{12}$$

Thus, the replicator dynamics equation of doctors can be written as $P_{1}\left(x\right)$ in Equation (1):


(1) $$P_{1}\left(x\right)=\frac{dx}{dt}=x\left(1-x\right)\left\{\begin{array}{l} y\left[r_{1}-c_{2}-\left(1-\alpha \right)c_{3}\right]-\\ c_{1}-\left(1-\beta \right)c_{4}+z\left(1-\beta \right)c_{4} \end{array}\right\}$$

When patients implement the “use” strategy, the marginal expected revenue is


$$\begin{array}{l} P_{21}=xz\left(e_{1}+e_{3}-h_{1}+L\right)+x\left(1-z\right)\left[\begin{array}{l} e_{1}+e_{3}-h_{1}-\left(1-\alpha \right)\\ h_{2}-\left(1-\beta \right)\left(h_{3}+h_{4}\right) \end{array}\right]\\ \;\;+\left(1-x\right)z\left(e_{2}\right)+\left(1-x\right)\left(1-z\right)\left[e_{2}-\left(1-\beta \right)h_{4}\right] \end{array}.$$

Meanwhile, when patients implement the “not use” strategy, the marginal expected revenue is


$$P_{22}=xz\left(e_{2}\right)+x\left(1-z\right)\left(e_{2}\right)+\left(1-x\right)z\left(e_{2}\right)+\left(1-x\right)\left(1-z\right)\left(e_{2}\right)$$

The expected revenue of the patients is


$$P_{2}\left(y\right)=yP_{21}+\left(1-y\right)P_{22}$$

Thus, the replicator dynamics equation of patients can be written as in Equation (2):


(2) $$P_{2}\left(y\right)=\frac{dy}{dt}=y\left(1-y\right)\left\{\begin{array}{l} x\left[\begin{array}{l} e_{1}-e_{2}+e_{3}-h_{1}-\\ \left(1-\alpha \right)h_{2}-\left(1-\beta \right)h_{3} \end{array}\right]\\ \begin{array}{l} +xz\left[\left(1-\alpha \right)h_{2}+\left(1-\beta \right)h_{3}+L\right]-\\ \left(1-\beta \right)h_{4}+z\left(1-\beta \right)h_{4} \end{array} \end{array}\right\}$$

When the platforms implement the “provide” strategy, the marginal expected revenue is


$$\begin{array}{l} P_{31}=xy\left(w_{1}-t_{1}-t_{2}+M\right)+x\left(1-y\right)\left(-t_{1}-t_{2}\right)\\ +\left(1-x\right)y\left(-t_{1}-t_{2}\right)+\left(1-x\right)\left(1-y\right)\left(-t_{1}-t_{2}\right) \end{array}.$$

However, when platforms implement the “not provide” strategy, the marginal expected revenue is


$$\begin{array}{l} \begin{array}{l} P_{32}=xy\left[\begin{array}{l} w_{1}-\alpha t_{1}-\beta t_{2}-\left(1-\alpha \right)\\ F_{1}-\left(1-\beta \right)\left(F_{2}+F_{3}+G\right)-N \end{array}\right]+\\ x\left(1-y\right)\left[-\alpha t_{1}-\beta t_{2}-\left(1-\beta \right)G\right] \end{array}\\ \begin{array}{l} +\left(1-x\right)y\left[-\alpha t_{1}-\beta t_{2}-\left(1-\beta \right)F_{3}\right]+\\ \left(1-x\right)\left(1-y\right)\left(-\alpha t_{1}-\beta t_{2}\right) \end{array} \end{array}.$$

The expected revenue of the platforms is


$$P_{3}\left(z\right)=zP_{31}+\left(1-z\right)P_{32}.$$

Thus, the replicator dynamics equation of platforms can be written as $P_{3}\left(z\right)$ in Equation (3):


(3) $$P_{3}\left(z\right)=\frac{dz}{dt}=z\left(1-z\right)\left\{\begin{array}{l} xy\left[\begin{array}{l} M+\left(1-\alpha \right)F_{1}+\\ \left(1-\beta \right)F_{2}+N \end{array}\right]-\left(1-\alpha \right)t_{1}\\ \begin{array}{l} -\left(1-\beta \right)t_{2}+x\left(1-\beta \right)\\ G+y\left(1-\beta \right)F_{3} \end{array} \end{array}\right\}.$$

### Stability analysis

The above three replication dynamic equations describe the dynamic adjustment process of the strategy selection of doctors, patients, and platforms. The game system has reached a stable state when the three groups continue to learn and imitate to reach the Nash equilibrium. To find the stability point of the evolutionary game among the three stakeholders, we assume:


$$\left\{\begin{array}{l} \frac{dx}{dt}=0\\ \frac{dy}{dt}=0\\ \frac{dz}{dt}=0 \end{array}\right\}.$$

Then, within the range of the equilibrium solution domain W={(x,y,z)|0≤x≤1;;0≤y≤1;;0≤z≤1|} are the following eight special equilibrium solutions: V0(0,0,0), V1(1,0,0), V2(1,1,0), V3(1,0,1), V4(0,1,0), V5(0,1,1), V6(0,0,1), V7(1,1,1). Another equilibrium point $E\left(x^{*},y^{*},z^{*}\right)$ is also in the above solution domain and satisfies:


(4) $$y\left[r_{1}-c_{2}-\left(1-\alpha \right)c_{3}\right]-c_{1}-\left(1-\beta \right)c_{4}+z\left(1-\beta \right)c_{4}=0$$

(5) $$x\left[\begin{array}{l} e_{1}-e_{2}+e_{3}-\\ h_{1}-\left(1-\alpha \right)\\ h_{2}-\left(1-\beta \right)h_{3} \end{array}\right]+xz\left[\begin{array}{l} \left(1-\alpha \right)h_{2}+\\ \left(1-\beta \right)h_{3}+L \end{array}\right]-\left(1-\beta \right)h_{4}+z\left(1-\beta \right)h_{4}=0$$

(6) $$\begin{array}{l} xy\left[M+\left(1-\alpha \right)F_{1}+\left(1-\beta \right)F_{2}+N\right]-\\ \left(1-\alpha \right)t_{1}-\left(1-\beta \right)t_{2}+x\left(1-\beta \right)G+y\left(1-\beta \right)F_{3}=0 \end{array}$$

Then, the three equations above are differentiated to obtain:


$$P_{1}^{\prime }\left(x\right)=\left(1-2x\right)\left[\begin{array}{l} y\left(r_{1}-c_{2}-\left(1-\alpha \right)c_{3}\right)-\\ c_{1}-\left(1-\beta \right)c_{4}+z\left(1-\beta \right)c_{4} \end{array}\right],$$

$$\begin{array}{l} P_{2}{}^{\prime }\left(y\right)=\left(1-2y\right)\{x\left[e_{1}-e_{2}+e_{3}-h_{1}-\left(1-\alpha \right)h_{2}-\left(1-\beta \right)h_{3}\right]\\ \begin{array}{ccc} & & +xz\left[\left(1-\alpha \right)h_{2}+\left(1-\beta \right)h_{3}+L\right]-\left(1-\beta \right)h_{4}+z\left(1-\beta \right)h_{4} \end{array}\} \end{array},$$

$$\begin{array}{l} P_{3}{}^{\prime }\left(z\right)=\left(1-2z\right)\{xy\left[M+\left(1-\alpha \right)F_{1}+\left(1-\beta \right)F_{2}+N\right]\\ \begin{array}{ccc} & & -\left(1-\alpha \right)t_{1}-\left(1-\beta \right)t_{2}+x\left(1-\beta \right)G+y\left(1-\beta \right)F_{3}\} \end{array} \end{array}.$$

According to the stability theorem of the evolutionary game, when $P_{1}{}^{\prime }\left(x\right)<0$,$P_{2}{}^{\prime }\left(y\right)<0$, $P_{3}{}^{\prime }\left(z\right)<0$in the above three formulas, x^*^, y^*^, and z^*^ represent the stable strategies that doctors, patients, and platforms should adopt in the evolution process.

#### Replicator dynamic analysis of the doctor group

According to equation (4),


$$y\left[r_{1}-c_{2}-\left(1-\alpha \right)c_{3}\right]-c_{1}-\left(1-\beta \right)c_{4}+z\left(1-\beta \right)c_{4}=0.$$

This equation represents the boundary of the steady state. When the following conditions are met


$$y\left[r_{1}-c_{2}-\left(1-\alpha \right)c_{3}\right]-c_{1}-\left(1-\beta \right)c_{4}+z\left(1-\beta \right)c_{4}>0,$$

then we obtain.

$P_{1}{}^{\prime }\left(0\right)>0$, $P_{1}{}^{\prime }\left(1\right)<0$.

This indicates that joining OHP is stable, and not joining OHP is unstable.

In contrast, when the following conditions are met.


$$y\left[r_{1}-c_{2}-\left(1-\alpha \right)c_{3}\right]-c_{1}-\left(1-\beta \right)c_{4}+z\left(1-\beta \right)c_{4}<0,$$

then we obtain.

$P_{1}{}^{\prime }\left(0\right)<0$, $P_{1}{}^{\prime }\left(1\right)>0$.

This indicates that not joining OHP is stable, and joining OHP is unstable. Whenx∈(0,1), $P_{1}\left(x\right)>0$. The phase evolution diagram of its stability depends on the shape of the quadratic curve of equation (4).

#### Replicator dynamic analysis of the patient group

According to equation (5),


$$\begin{array}{l} x\left[e_{1}-e_{2}+e_{3}-h_{1}-\left(1-\alpha \right)h_{2}-\left(1-\beta \right)h_{3}\right]+\\ xz\left[\left(1-\alpha \right)h_{2}+\left(1-\beta \right)h_{3}+L\right]-\left(1-\beta \right)h_{4}+z\left(1-\beta \right)h_{4}=0. \end{array}$$

This equation represents the boundary of the steady state. When the following conditions are met


$$\begin{array}{l} x\left[e_{1}-e_{2}+e_{3}-h_{1}-\left(1-\alpha \right)h_{2}-\left(1-\beta \right)h_{3}\right]+\\ xz\left[\left(1-\alpha \right)h_{2}+\left(1-\beta \right)h_{3}+L\right]-\left(1-\beta \right)h_{4}+z\left(1-\beta \right)h_{4}>0, \end{array}$$

then we obtain.

$P_{2}{}^{\prime }\left(0\right)>0$, $P_{2}{}^{\prime }\left(1\right)<0$.

This indicates that using online medical treatment is stable, and not using online medical treatment is unstable.

In contrast, when the following conditions are met


$$\begin{array}{l} x\left[e_{1}-e_{2}+e_{3}-h_{1}-\left(1-\alpha \right)h_{2}-\left(1-\beta \right)h_{3}\right]+\\ xz\left[\left(1-\alpha \right)h_{2}+\left(1-\beta \right)h_{3}+L\right]-\left(1-\beta \right)h_{4}+z\left(1-\beta \right)h_{4}<0, \end{array}$$

then we obtain.

$P_{2}{}^{\prime }\left(0\right)<0$, $P_{2}{}^{\prime }\left(1\right)>0$.

This indicates that not joining online medical treatment is stable, and joining online medical treatment is unstable. When x∈(0,1), $P_{2}\left(x\right)>0$. The phase evolution diagram of its stability depends on the shape of the quadratic curve of equation (5).

#### Replicator dynamic analysis of the platform group

According to equation (6),


$$\begin{array}{l} xy\left[M+\left(1-\alpha \right)F_{1}+\left(1-\beta \right)F_{2}+N\right]-\\ \left(1-\alpha \right)t_{1}-\left(1-\beta \right)t_{2}+x\left(1-\beta \right)G+y\left(1-\beta \right)F_{3}=0 \end{array}$$

This equation represents the boundary of the steady state. When the following conditions are met,


$$\begin{array}{l} xy\left[M+\left(1-\alpha \right)F_{1}+\left(1-\beta \right)F_{2}+N\right]-\\ \left(1-\alpha \right)t_{1}-\left(1-\beta \right)t_{2}+x\left(1-\beta \right)G+y\left(1-\beta \right)F_{3}>0, \end{array}$$

then we obtain.

$P_{3}{}^{\prime }\left(0\right)>0$, $P_{3}{}^{\prime }\left(1\right)<0$.

This indicates that using OHP is stable, and not using OHP is unstable.

In contrast, when the following conditions are met,


$$\begin{array}{l} xy\left[M+\left(1-\alpha \right)F_{1}+\left(1-\beta \right)F_{2}+N\right]\\ -\left(1-\alpha \right)t_{1}-\left(1-\beta \right)t_{2}+x\left(1-\beta \right)G+y\left(1-\beta \right)F_{3}<0 \end{array},$$

then we obtain.

$P_{3}{}^{\prime }\left(0\right)<0$, $P_{3}{}^{\prime }\left(1\right)>0$.

This indicates that not joining OHP is stable, and joining OHP is unstable. When x∈(0,1), $P_{3}\left(x\right)>0$. The phase evolution diagram of its stability depends on the shape of the quadratic curve of equation (6).

## Simulation analysis

To explore the evolution of OHP under different parameter values, based on the established evolutionary game model, we analyze the platforms’ QISC, IPISC, the initial state of doctors and patients, the doctor’s registration costs, time costs, and reputation loss, and the patients’ online healthcare costs. To determine the simulation parameters, we refer to the relevant data on the Spring Rain Doctor platform, consult experts who study simulation in related fields, and combine relevant literature research. These simulation parameters can reflect the general trend to some extent.

### The influence of QISC α on the OHP evolutionary game behavior

**Figure 1:**
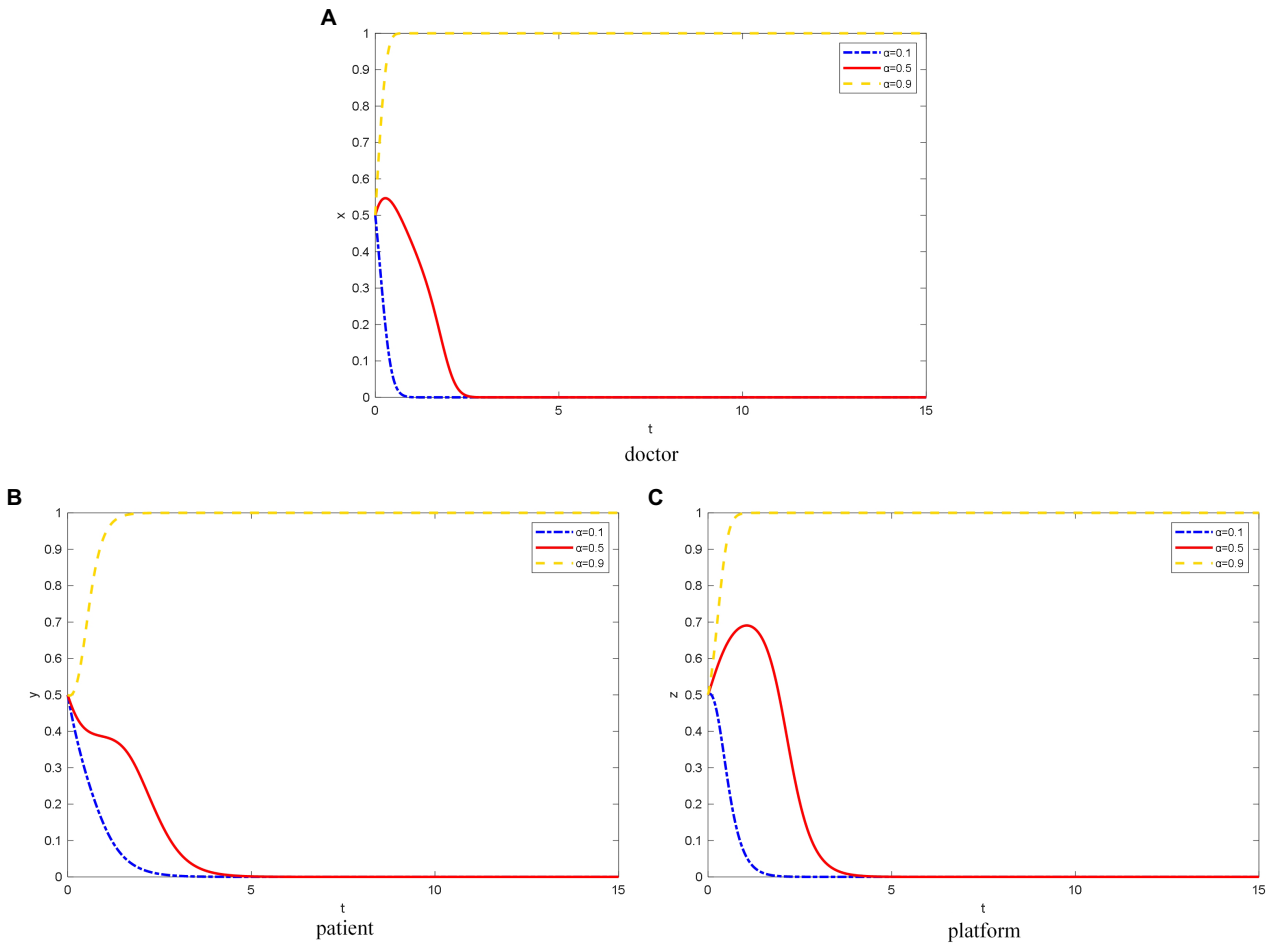
The evolution path of different objects’ strategy under different QISC. **(A)** Doctor, **(B)** patient, **(C)** platform.

α takes the value of 0.1, 0.5, and 0.9 for low, medium, and high QISC, respectively. As shown in Figure 1A, doctors doubt the credibility of the platform due to the platforms’ medium and low QISC, and they tend to use the strategy of not joining OHP. The lower the QISC, the faster the rate of evolution to the strategy of not joining. When the platform has a higher degree of QISC, doctors tend to believe the operation level of the platform. Therefore, OHP can improve the healthcare efficiency of doctors and their additional income. Eventually, doctors will tend to join OHP.

As shown in Figure 1B, when the platforms’ QISC is low, patients doubt the healthcare level of the doctors on the platform and the reliability of the platform based on the doctors’ information. Due to the fear of misdiagnosis, the patients are likely to choose the strategy of not using OHP. When the platforms’ QISC is moderate, the patients initially tend to not use OHP due to the psychology of observation. However, after patients can accurately judge the qualifications of doctors through the patient and peer evaluation mechanism on the platform, they exhibit a tendency to use OHP.

When the platforms’ QISC is high, patients can learn about the doctors’ healthcare level through the doctor-related information published on the platform, such as the medical institution, region, and professional title. Because patients can save time and money by using OHP, they can enjoy healthcare resources that were not previously available due to several factors such as region and income level. Therefore, patients will tend to use OHP.

As shown in Figure 1C, when the platforms’ QISC is low, the platforms’ inspection of doctors and its construction are relatively negative. Thus, the platform tend to not provide standardized online healthcare services.

Meanwhile, when the platforms’ QISC is moderate because the platforms consider reputation, they will invest part of the funds to build the platforms’ qualification inspection and information protection mechanism. However, over time, the platforms’ revenue is not as expected, and the platforms do not pay much attention to the brand and popularity. Eventually, platforms tend to use the strategy of not providing standardized online healthcare services.

However, when platforms have a high degree of QISC, they pay more attention to its own construction, social reputation, and long-term development. They invest heavily in doctor qualification inspection and information protection, and they are willing to take certain risks. Thus, the platforms eventually tend to provide standardized online healthcare services.

### The influence of IPISC β on OHP evolutionary game behavior

*β* takes the value of 0.1, 0.5, and 0.9 for low, medium, and high IPISC, respectively.

**Figure 2:**
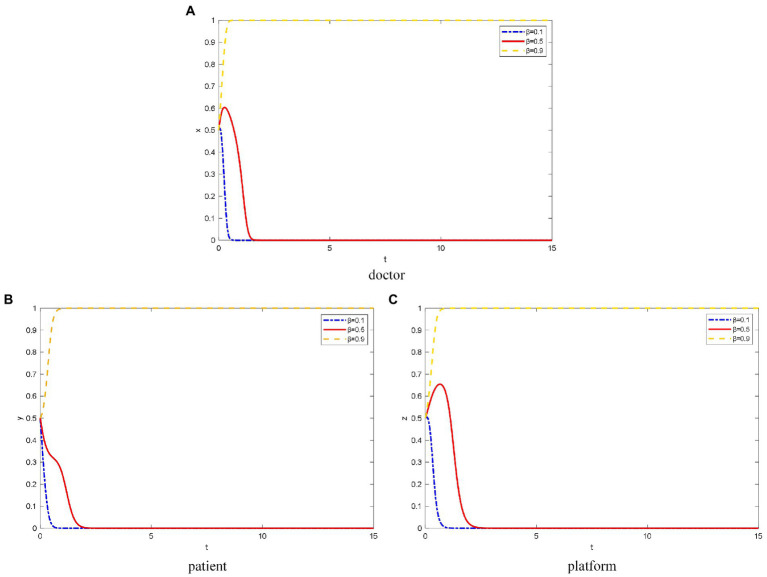
The evolution path of different objects’ strategy under different IPISC. **(A)** Doctor, **(B)** patient, **(C)** platform.

As shown in Figure 2A, when the platforms’ IPISC is low, doctors concern about their own information security, which will lead to the evolution of not joining OHP.

However, when the platforms’ IPISC is moderate, doctors will choose to join OHP for a certain period to observe the quality of the platforms’ operation. However, the platforms’ investment in information protection cannot meet the needs of doctors for their information security protection. Thus, the doctors finally choose not to join the OHP.

When the platforms invest heavily in information protection, doctors elect to trust the platforms’ information protection mechanism and join OHP. In addition, the greater the IPISC, the faster doctors elect to join OHP.

For patients, when the IPISC is low, they are concerned about the leakage of their private and identity information, and they tend to not use OHP (Figure 2B). The lower the platforms’ IPISC, the faster the patients choose not to use it. When the platforms’ IPISC is moderate or high, patients elect to trust the platforms’ information protection mechanism and choose to use OHP.

As shown in Figure 2C, when platforms’ IPISC is low, they lack attention to the construction of information protection mechanisms. Thus, the platforms ultimately choose not to provide standardized online healthcare services.

In the case of moderate investment in platform information protection, the platforms initially elect to provide standardized online healthcare services because they want to attract doctors and patients. Over time, after a large number of doctors and patients join and use OHP, respectively, the platform thinks investing heavily in doctor qualification inspection and information protection is not necessary because of the cost. Thus, platforms ultimately choose not to provide standardized online healthcare services.

When the platforms’ IPISC is high, they pay more attention to the protection of doctors’ and patients’ privacy information and invest more funds to protect it. At this time, the platforms care more about long-term interests, so they choose to provide standardized online healthcare services. The higher the platforms’ IPISC, the faster they choose to provide standardized online healthcare services.

### Influence of the different initial states of doctors on the OHP evolution game behavior

**Figure 3:**
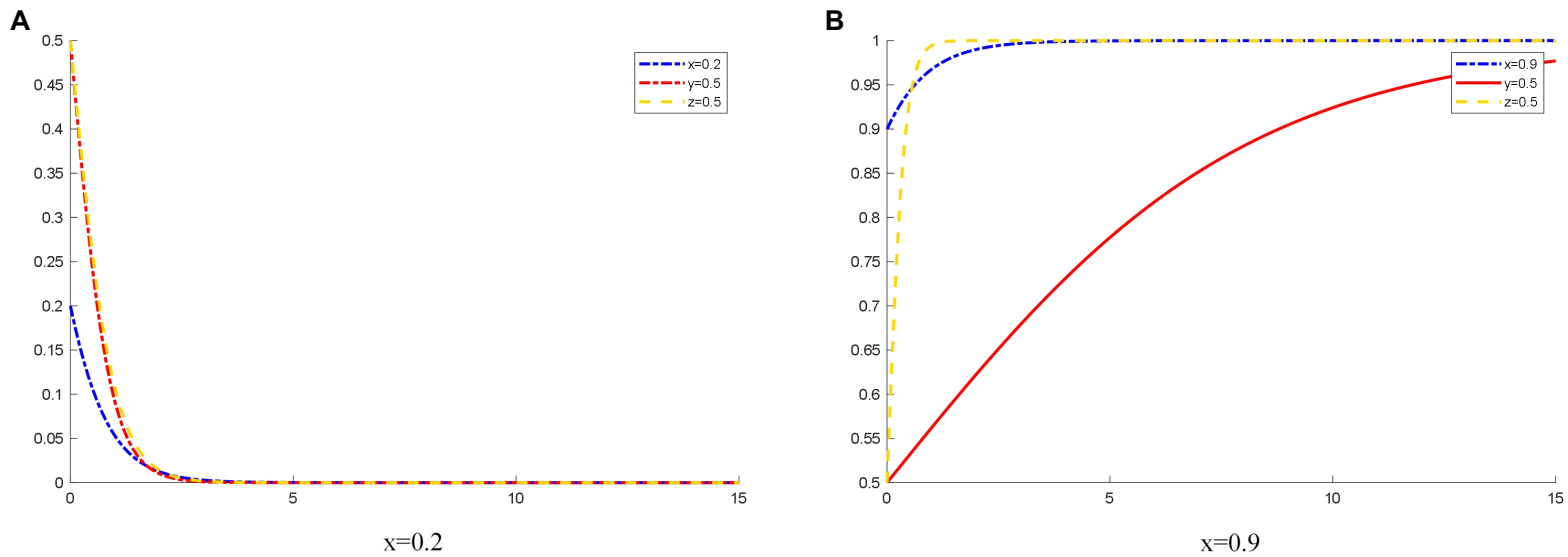
The evolution track of three-party strategy selection under different initial states of doctors. **(A)** *x* (1) = 0.2; **(B)** *x* (1) = 0.9.

As shown in Figure 3, when the probability of doctors’ initial choice to join the OHP is 0.2, doctors’ attitude toward joining the OHP is negative. Therefore, patients concern about OHP and doubt the reliability of the platform. Eventually, the patients elect not to use OHP. Because doctors do not join OHP, platforms cannot attract patients, and they quickly choose not to provide standardized online healthcare services.

Meanwhile, when the probability that the doctors initially choose to join OHP is 0.9, the doctors’ willingness to join OHP is relatively strong. Moreover, many doctors signed the contracts with the platform. The platform has rich medical resources. These resources enable patients to trust the platforms, so they choose to use OHP. At this time, the platform has good thinking, and they choose to provide standardized online healthcare services much faster than patients choose to use OHP.

### Influence of the different initial states of patients on the OHP evolution game behavior

**Figure 4:**
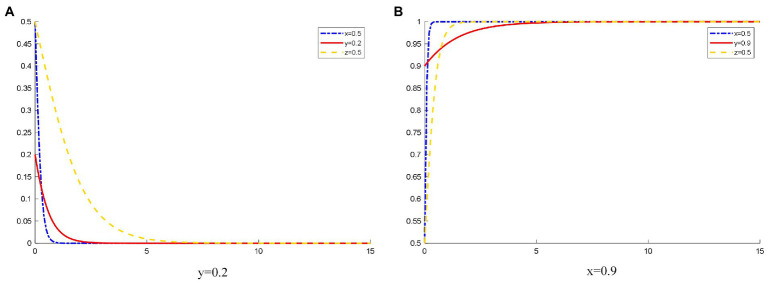
The evolution track of three-party strategy selection under different initial states of patients. **(A)** *y* (1) = 0.2; **(B)** *y* (1) = 0.9.

As shown in Figure 4, when the probability that the patients initially choose to use OHP is 0.2, the patients’ willingness to use OHP is low due to the platforms’ operation, publicity, and other factors. OHP has not been popularized in society. The platform lacks a large number of users. At this time, the platform is reluctant to spend too much cost on operating online healthcare services and tends to not provide standardized online healthcare services.

When the probability that patients initially choose to use OHP is 0.9, most patients choose to use OHP. This form of OHP is popular among the public. At this time, a large amount of online medical resources is required. A large number of patients choosing to use OHP would improve the medical efficiency of doctors and reduce the pressure on hospitals. Doctors can also obtain additional income. Therefore, doctors choose to join OHP. Moreover, a large number of users registered on the platform provide opportunity for the platforms to obtain huge profits. They further improve the quality of platform services for their reputation and patients, strictly inspect the qualifications of doctors, and actively protect the safety of patient information. Eventually, platforms tend to provide standardized online healthcare services.

### Influence of different registration costs on the evolutionary behavior of doctors

**Figure 5:**
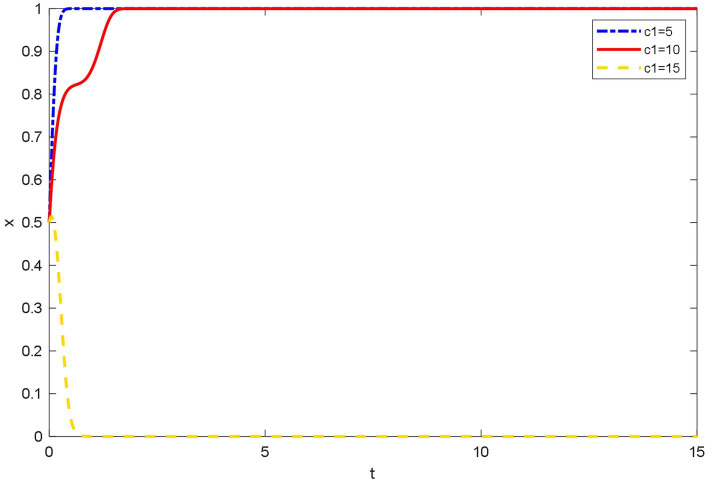
Evolutionary trajectory of doctor’s strategy selection under different registration costs.

As shown in Figure 5, when the qualification registration costs are high, doctors tend not to join OHP. Doctors are faced with the difficulty of registration. When inspecting the qualifications of doctors, the platforms require doctors to upload qualification and work certificates. Some doctors think that registration and cancelation procedures are cumbersome. Therefore, they ultimately tend not to join OHP. However, when the qualification registration costs are low, doctors tend to join OHP. This reflects that doctors’ satisfaction and joining costs are important to the joining behavior of doctors.

### Influence of different time costs on the evolutionary behavior of doctors

**Figure 6:**
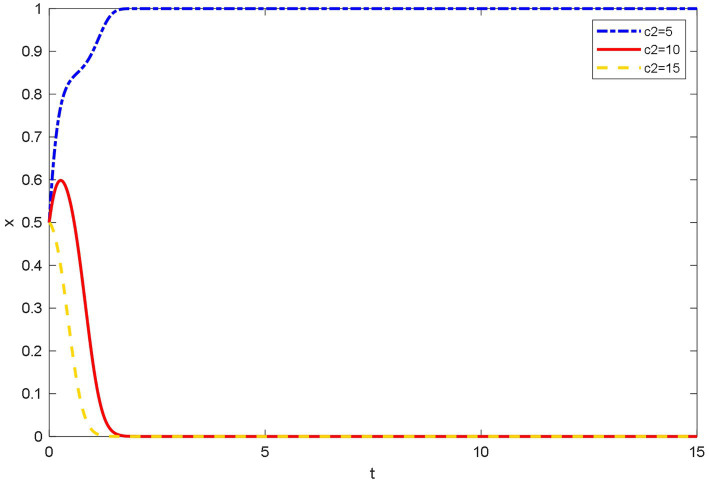
Evolutionary trajectory of doctors’ strategy selection under different time costs.

Figure 6 shows that when the time costs are low, doctors are willing to use their free time to join OHP to increase their income and reputation. Doctors can have online medical treatment any time, which is why doctors choose to join OHP. When the time costs are medium, at first, doctors try to choose joining OHP, and then because of lack of time and energy, they finally elect not to join OHP. However, they tend to do their jobs in the hospital when the time costs are high. At this time, they are unwilling to spend too much time on OHP and ultimately choose not to join OHP.

### Influence of different reputation loss on the evolutionary behavior of doctors

**Figure 7:**
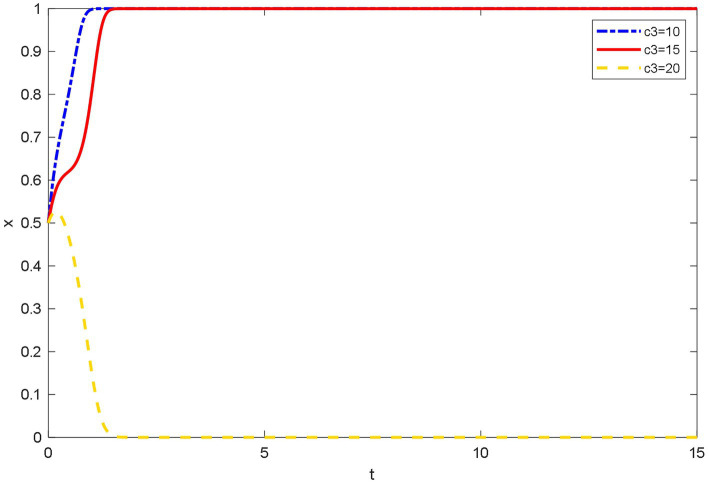
Evolutionary trajectory of doctors’ strategy selection under different reputation loss.

Figure 7 shows that the reputation loss of doctors among patients is small because the evaluation mechanism of patients on doctors set by the platforms is more reasonable and objective, and the professional level and working attitude of doctors can be evaluated fairly. At this time, the doctor has a greater willingness to join OHP. When the doctors’ reputation is greatly lost, in addition to the medical level, the patients’ requirements for the doctors’ service level are also higher, and the doctor is more likely to receive negative evaluations about communication skills, timeliness, and service attitudes. For example, the experience mechanism of doctors will be questioned by patients; patients are more willing to trust the opinions of doctors in offline hospitals than OHP. Moreover, due to the availability of online knowledge, patients’ psychological expectations for OHP are too high, which will lead to patient’s provision of negative comments. Initially, doctors choose to join OHP. Over time, negative online reviews will put pressure on doctors, and they ultimately choose not to join OHP.

### Influence of different online healthcare costs on the evolutionary behavior of patients

**Figure 8:**
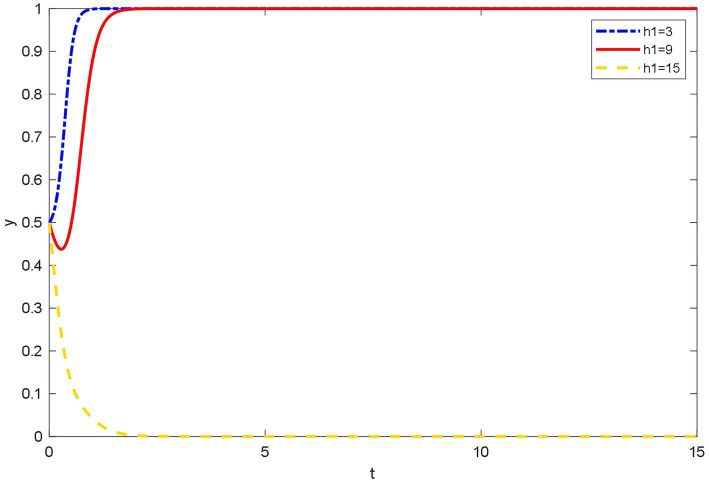
Evolutionary trajectory of patient’s strategy selection under different online healthcare costs.

As shown in Figure 8, when the online healthcare costs are low, using OHP can save patients time and money to go to the hospital for medical treatment. This is because many patients’ conditions can be solved by simple diagnosis and treatment. Especially, patients can reduce the costs of useless consultations by using OHP, and OHP can solve the problems of difficult and expensive medical treatment.

Patients tend choosing not to use OHP initially when the online healthcare costs are moderate. This is because at the beginning, people’s awareness of paying for knowledge was not strong. However, with time, people’s health and consumption concepts have improved. The concept of obtaining high-quality healthcare services by paying lower medical expenses than that of hospitals has gradually become popular among patients. Eventually, patients elect to use OHP.

When online healthcare costs are high, patients tend to not trust the platforms and doctors even though OHP has been rapidly promoted and patients have gradually accepted this medical method due to the convenience. Many factors make patients doubt the professionalism of doctors and the reliability of platforms. When OHP cannot help patients save the cost of seeing a doctor, patients tend not to use OHP.

## Case analysis: An example of spring rain doctor platform

Spring Rain Doctor, a developing platform for nearly 8 years since 2011, is the first Internet enterprise in China to try online healthcare. More than 300 public hospitals have settled on the platform. By the end of August 2020, the platform has 630,000 Chinese practicing doctors from all departments. The cumulative number of users of the platform is 130 million, and its daily consulting times exceed 300,000. It is the world’s largest mobile doctor–patient communication platform. Doctors’ communication with patients is conducted through the website, pictures, mobile phones, and even video format. In addition, doctors could observe timely feedback and service assessments of patients (Chen et al., 2020).

According to Artery Network data, Table 2 shows the proportion of platform users in each region. Guangdong has the largest number of users, accounting for 11.2%, far more than other regions; followed by Shandong, accounting for 7.16%. The reason for this phenomenon may be that Guangdong is a young city, and people are more interested in new things and are willing to try new medical methods. Moreover, the *per capita* income in the region is relatively high, and people’s time and opportunity costs are huge. If users can consult online at any time and save the time and cost of going to the hospital, they will be more willing to pay for consultation on the platform. As shown in Table 3, eight of the top ten provinces with the number of users of the Spring Rain Doctor platform are among the top ten provinces by population in 2018. It can be inferred that the population of Spring Rain doctors is evenly distributed across the country, and the population of platform users is not restricted by region. This shows that various regions have the problem of a lack of medical resources. Different regions have different problems. For example, developed regions have more medical resources, but the number of patients is large, and it is difficult to see a doctor. In rural areas, medical resources are scarce, and it is expensive for patients to see a doctor.

**Table 2:** Proportion of users in each province.

Province	The proportion of users in each province (%)
Guangdong	11.2
Shandong	7.16
Hebei	6.05
Jiangsu	5.85
Sichuan	5.45
Zhejiang	4.94
Henan	4.94
Liaoning	4.54
Heilongjiang	4.04
Hubei	3.43

**Table 3:** The top 10 provinces in population, 2018.

	Province	Population (million)
1	Guangdong	111.69
2	Shandong	100.05
3	Henan	95.59
4	Sichuan	83.02
5	Jiangsu	80.29
6	Hebei	75.19
7	Hunan	68.60
8	Anhui	62.54
9	Hubei	59.02
10	Zhejiang	56.57

**Figure 9:**
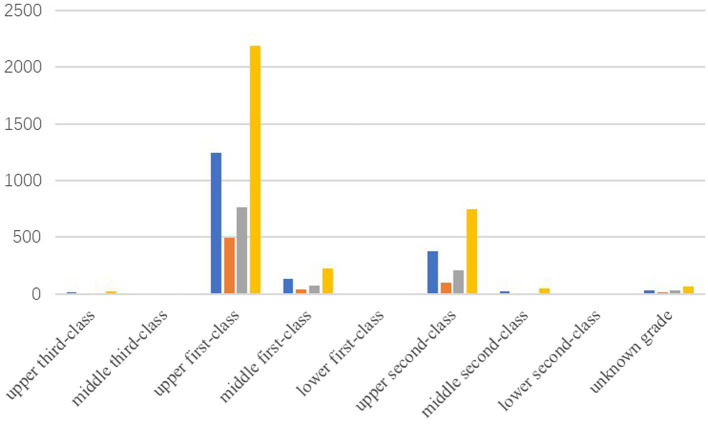
Number of online doctors at all levels.

Online healthcare services are one of the “standard configurations” of various mobile medical enterprises. This is also one of the most competitive areas. However, only the most reliable medical resources are the most powerful competitive forces. In response, Dr. Wang Jianguo, Vice President of Spring Rain Doctor, released the “Online Diagnosis and Treatment Ability Report” at the meeting. Spring Rain Doctor solves more than 330,000 health problems daily, with an average response time of about 3 min. The source of doctors covers all provinces (autonomous regions, municipalities directly under the Central Government) except Hong Kong, Macao, and Taiwan. Most doctors belong to the 25–45 years age group: senior doctors account for 24%, and other doctors are 38%. In terms of doctor qualifications, Spring Rain Doctor has a strict inspection mechanism. First, it requires four cards, namely, doctor’s qualification certificate, a practicing qualification certificate, an ID card, and a bank card showing their real-name information. Then, staff will confirm the doctor’s work by phone or offline to determine if the doctor is indeed a working doctor. Simultaneously, the most active and largest group of Spring Rain Doctor comes from the upper first-class hospitals. Figure 9 shows the number of online doctors from the upper first-class hospitals based on the data provided by Artery Network: there are 4,680 doctors, accounting for 62.3% of the total online doctors. This means that the Spring Rain Doctor platform has a large number of high-quality medical resources. In addition, when the doctor serves online, he/she will undergo a strict evaluation mechanism. This shows that the platform’s inspection of doctor qualifications is an important factor affecting the platform’s online healthcare competitiveness. The findings in Figure 1 are verified.

On the protection of privacy information, the General Office of the State Administration for Market Regulation, the Secretariat of the Cyberspace Administration of China, Ministry of Industry and Information Technology of the People’s Republic of China, and the General Office of the Ministry of Public Security jointly issued the Method for Determining the Illegal Collection and Use of Personal Information by mobile apps on December 30, 2019. Spring Rain Doctor and 10 other apps were reported for illegally collecting and using personal information because they did not clarify to the users to apply for all privacy rights. Users ask for the privacy of their diseases on the Spring Rain Doctor platform to avoid being discovered by acquaintances. After that, the Spring Rain Doctor app launched a privacy policy. Therefore, the protection of the platform’s information is an important factor for patients choice of using OHP. If the platform violates the privacy of patient users, it will result in the loss of a large number of users. Thus, the findings in Figure 2 are verified.

In 2016, Spring Rain Doctor, as the largest online consultation platform in the country, sets up the online consultation service function for hardware manufacturers, APPs, websites, WeChat accounts, etc. who have this demand for free. After the opening of Spring Rain Doctor’s online consultation platform, a borderless online consultation portal was used to dig deeper into the platform’s online consultation capabilities, strengthen the “foundation” role of this service for mobile healthcare and strive to become the second entrance to health issues of Chinese. With the expansion of Spring Rain Doctor’s online healthcare portal, patients can use online healthcare functions more conveniently, and the popularization of its online healthcare functions can be promoted consequently. This shows that Spring Rain Doctor promotes online healthcare as much as possible to increase the probability of patients using OHP. Thus, the findings in Figure 4 are verified.

The doctors were initially interviewed one by one by Spring Rain Doctor’s offline team to obtain medical resources. After the brand is being promoted, the number of doctors added through the brand effect exceeded the number of doctors promoted. To obtain a large number of doctor resources, and thus avoid shortage problem of medical resources, Spring Rain Doctor allocates different patients to doctors by layering the needs of users or profiles doctors with different capabilities and services on different platforms. Thus, the doctor and patient can obtain a good match. Simultaneously, to persuade doctors to join, Spring Rain Doctor reduces the registration cost. Spring Rain Doctor’s registration and auditing are more user-friendly, especially auditing, supporting everyone to use badges and work permits to replace qualification certificates and some complicated proofs. This reduces the doctor’s registration costs and difficulty of usage, while ensuring the authenticity of the doctor’s qualifications. This shows that reducing the registration costs of doctors is also a way for the platform to attract doctors to join. Hence, we verified the findings in Figure 5.

Table 4 presents the income of doctors on the Spring Rain Doctor platform as of September 2014 based on the Artery Network data. The doctor’s income is divided into two parts. One part is the income obtained by answering users’ free consultations, which is paid by the Spring Rain Doctor platform at 1.5 yuan for each reply. The second part is the dynamic pricing of doctors, which is paid by users. Note that since the dynamic pricing part of doctors’ income has been changing, the gap between the data and the doctor’s actual income may be large. However, it can be seen that the platform attracts more doctors to join through subsidies. This will make the doctors joining the Spring Rain Doctor platform more active and attract a large number of users. In summary, the Spring Rain Doctor platform uses various methods to increase the probability of doctors joining. Thus, the findings in Figure 3 are verified.

**Table 4:** Income of each department on Spring Rain Doctor platform.

Department	Income (million)
Pediatrics	2.48
Otolaryngology	0.56
Obstetrics and Gynecology	2.98
Orthopedics	0.68
Stomatology	0.34
Male Urology	1.56
Cranial Nerve	0.85
Endocrinology	0.45
Internal Medicine	3.84
Dermatology	1.48
Surgery	2.30
Psychology	0.54
Cardiovascular	0.75
Ophthalmology	0.42
Nutritional	0.14
Plastic Surgery	0.40
Chinese Medicine	0.52
Oncology	0.46
Grand Total	20.76

The most important purpose for doctors to join OHP is to increase economic and reputational benefits. Through research, the doctors on the platform of Spring Rain Doctor report that the platform arbitration is biased toward users when disputes occur between doctors and patients. Spring Rain Doctor’s doctor evaluation system drop more points than other platforms. It is often the case that doctors did not respond in time and receive bad reviews. These problems have dampened the enthusiasm of doctors. The common point of these problems is that they damage the reputation of doctors. Therefore, the loss of the reputation of doctors is an important factor for doctors to consider joining OHP or not. Thus, the findings in Figure 7 are verified.

**Figure 10:**
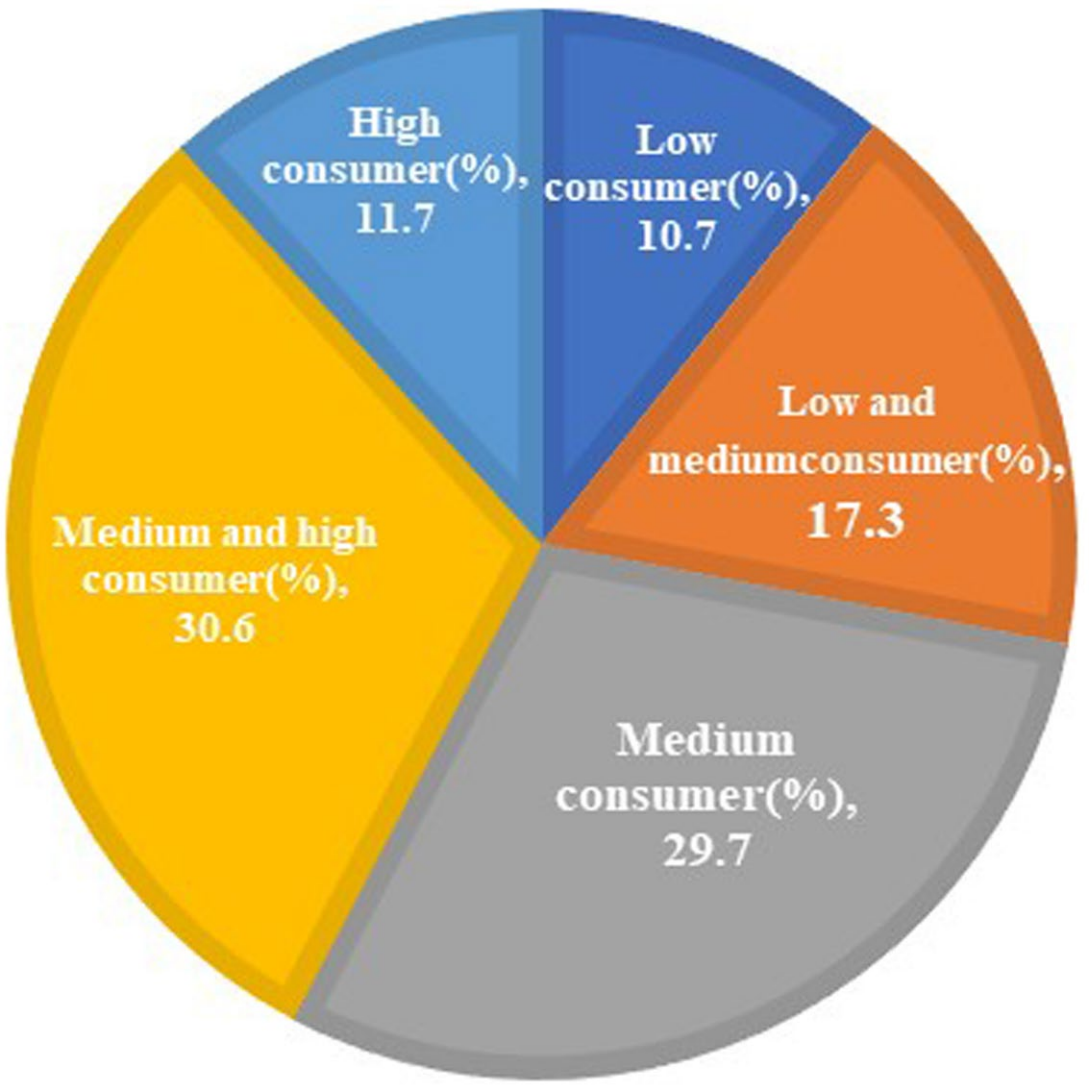
Analysis on the consumption ability of Spring Rain Doctor’s users.

Figure 10 shows that the users of Spring Rain Doctor platform are concentrated in the middle of the consumption level. This shows that OHP is more acceptable to the public. However, users below the medium consumption level account for more than 50% of all users. This shows that online healthcare costs are still a key factor for patients to use the Spring Rain Doctor platform. Hence, the findings in Figure 8 are verified.

According to the user survey report of Spring Rain Doctor platform (data from www.woshipm.com), an in-depth interview with doctors is conducted. Examples of survey questions and interview results are shown in Table 5. As shown in Table 5, income and word of mouth are the main motivations for doctors to join OHP, and time cost is the main factor affecting doctors’ joining behavior. The findings in Figure 6 are verified.

**Table 5:** Examples of survey questions and interview results.

Question	Result
Do you provide private doctor services?	We can provide the services of a private doctor when we have the time and energy. However, we do not want to take up too much working time.
What is your motivation for online health services?	We can use our free time to increase revenue and reputation.
Have you ever received negative reviews when providing online health services?	The main reason we received bad reviews was that we did not respond in time.
What are your requirements and suggestions for the platform?	Users can clearly explain the complete symptoms at one time during the consultation, thereby saving time.

Based on the above analysis, this article uses the case of Spring Rain Doctor platform to verify the simulation results. This further indicates that the research results are reasonable and have strong practical significance, which is in line with the specific situation of the development of OHP in China.

## Discussion

### Theoretical implication

We expand and complement the literature on online healthcare. The theoretical implication of this article is as follows.

First, previous studies have explored the factors that affect patients’ use of OHP based on the perspectives of PVT (Dong, 2019), social support theory (Tang et al., 2018), PCT (Sun et al., 2022), and UTAUT (Nunes et al., 2019). Some studies have also explored the influencing factors of doctors’ participation in OHP based on the perspectives of SDT (Yang et al., 2019c), social capital theory (Lin et al., 2016) and HAPA (Liu and Yu, 2022). However, there is no research that integrates PVT, PCT, and SDT. Based on the above theories, this article incorporates the involved influencing factors into the evolutionary game model, deeply explores the evolutionary mechanism of the behavior of doctors, patients and platforms, further verifies the previous research results, expands the research perspective and theoretical system of online healthcare.

Secondly, previous studies have used evolutionary game theory to explore the behavior of doctors and patients (Chen et al., 2018). Some scholars also constructed an evolutionary game model with users and online health communities as the main participants (Xu et al., 2022). However, no study has constructed an evolutionary game model with doctors, patients and platforms as participants, and explored the adoption conditions and promotion strategies of OHP through the evolutionary game model. This article establishes a tripartite evolutionary game model of doctors, patients and platforms, analyzes the evolutionary stability strategy, and explores the influence of different factors on the evolutionary stability strategy.

Finally, this article combines the theoretical model, numerical simulation and case analysis, and uses the actual case of Spring Rain Doctor to verify the rationality of the model and simulation results. This is of great theoretical significance to the research on OHP application and promotion strategies in China.

### Practical implication

This article establishes a game model for the evolution of the players in the online medical field based on the premise of the bounded rationality of the game party. Through the analysis of the three-party evolution game model and the numerical simulation analysis of the evolutionary behavior of doctors, patients, and platforms, we found that the higher the platforms’ inspection of doctor qualifications, the more it can strengthen doctor’s and patient’s trust and promote their participation and use of the platform. Because of the low and medium intensity scrutiny, doctors and patients will choose not to join or use the platform for fear of misdiagnosis. Based on this conclusion, the platform should establish a strict and perfect qualification review mechanism, conduct standardized training, clarify the platform operation standards for doctors, and carry out legal risk education for online healthcare. On the one hand, this can avoid the misdiagnosis of patients, so that patients can get a good consultation experience. Previous studies have proved that the higher the quality of the physician, the higher the levels of patient satisfaction (Wei and Hsu, 2022). On the other hand, the platform can build a brand image and enhance doctors’ trust in the platform, which can enable more doctors to participate in OHP. Especially in China, some doctors in second-class hospitals and third-class hospitals have more spare time. The platform can improve the review mechanism to attract these doctors to join, thereby releasing redundant medical resources and reducing the pressure of medical treatment in areas with severe epidemics. Doctors can also gain opportunities for self-improvement, increase reasonable income, and build personal brands. At the same time, the platform can also cover various regions of China more widely.

When the platforms’ privacy information protection is moderate or low, doctors and patients tend to choose not to join or use OHP. The greater the protection of the platforms’ information, the more it is able to promote doctors and patients to join and use OHP. Indeed, evidence in the literature indicates patients’ hesitation to disclose their personal information online; hence, they switch doctors frequently or switch to an offline hospital visit (Yang et al., 2019b). Thus, the platforms’ information protection mechanism is an important factor in doctors’ and patients’ choice of joining or using OHP. The competent government department should clarify the entry threshold for OHP as soon as possible, improve the information security management system, ensure user information security and privacy, and better play the role of OHP. At the same time, different from previous research conclusions, we believe that privacy information protection not only affects patients’ willingness to use, but also affects doctors’ participation. We believe that this is because doctors may be harassed by patients due to information leakage, thereby affecting their normal work and life. The platform should improve the security and reliability of the system, and improve the ability to resist hacker attacks. This can not only improve patients’ willingness to use OHP, but also encourage doctors to actively participate in OHP by protecting their personal information.

For patients, the richer the medical resources and the higher the quality of the platforms, and the lower the costs of OHP, the more the patients are encouraged to use OHP, thus saving more time and money costs. Previous studies have confirmed that online consultation can provide patients with convenient access to physicians at low cost. Therefore, the platforms should vigorously promote OHP, so that more doctors can actively join in, thereby attracting more patients. OHP can be promoted in many ways, but grassroots doctors are the entrance with the highest conversion rate. Grassroots doctors can connect medical resources and patients to a certain extent, which will be a key part of the cross-border integration of the Internet and the medical industry. The platform should concentrate high-quality resources on grassroots doctors, provide them with certain training opportunities and room to learn, and increase their income.

Meanwhile, the stronger the patients’ willingness to use OHP, the more inclined the doctors are to join OHP, thereby obtaining more benefits. This proves that the user traffic of the platform is an important influencing factor for doctors to participate in OHP. According to SDT, doctors are attracted by the economic and social benefits of large numbers of patients (Yang, 2019). Therefore, the core of OHP promotion is the patient. For China, remote cities and township residents are the largest customer groups of OHP. The OHP is just a new form of service in the medical industry. Hence, promoting the popularization of OHP in remote and township residents can reflect the true value of OHP and thus promote the development of the entire mobile medical industry. Therefore, the platforms should increase publicity and advertising in remote cities and rural township residents to attract more medical workers to join the OHP field.

Simultaneously, registration costs, time costs, and reputation loss affect doctors’ participation in OHP. Based on PVT, we argue that registration costs can affect physicians’ perceived ease of use. The platform should simplify the registration process and make it more user-friendly and convenient. At the same time, if a doctor provides a satisfactory service online, it may simultaneously help him/her gain reputation from both online and offline channels through word of mouth communication (Chen et al., 2020). According to trust theory, doctor’s reputation reflects the quality of doctor’s service and will have an impact on patient’s choice (Gong et al., 2021). Therefore, the loss of a doctor’s reputation will harm the doctor’s enthusiasm to participate in OHP. Different from previous research conclusions, this paper believes that the reputation loss of doctors comes not only from the negative comments of patients, but also from the unreasonable reputation evaluation mechanism of the platform. Meanwhile, when doctors decide to offer online counseling services in their free time, the number of consultations and extra devoted time is considered negative factors affecting doctors’ initiatives (Chen et al., 2020). Without proper stimulating motivators, joining in OHP is stressful for doctors because they have heavy work in hospitals (Yang et al., 2019c). The platform should optimize the function of operation process and add online and offline status for doctors in the graphical consultation interface, so that patients will not give bad reviews to doctors because of the long waiting time. Specifically, the platform should add functions such as message withdrawal, message copying, sending small videos, voice-to-text, and service end countdown, increase the number of remaining conversations in the dialog boxes of both parties and the reply status of the other party, and reduce the upper limit of the number of conversations within the service time limit. We suggest that platforms should use technology to improve the communication efficiency between doctors and patients, reduce the reputation loss and time cost of doctors, and make rational use of limited and valuable medical resources.

## Limitations and future research

Although we found some implications of OHP promotion, much work is still needed in the future. For example, we can study the behavior of OHP subjects from the perspective of government regulation and whether free consultation will affect the enthusiasm of doctors. In the future, the government must be included into the scope of OHP subjects to further study the promotion of OHP.

Futhermore, this article does not consider the lags of earnings and returns, as well as the issue of organizational externalities. Future research should consider time as a factor and include the influencing factors of organizational externalities.

Moreover, with the development of Internet digitalization, cutting-edge technologies such as big data, artificial intelligence, blockchain, cloud computing and 5G have unique advantages in the field of digital information. The integration of online healthcare and cutting-edge technology is an inevitable trend. Online healthcare based on emerging technologies not only improves the efficiency of diagnosis and treatment, but also brings a series of risks such as data privacy and accountability. Therefore, future research can combine the characteristics of digital technology to discuss the promotion strategy and safeguard measures of online healthcare.

## Conclusion

In this article, through the establishment of an evolutionary game model of OHP stakeholders, we found that the platforms’ qualification inspection of doctors, investment in information protection, initial probabilities of doctors joining and patients using the platform, doctors’ registration and time costs and reputation loss, and patients’ online healthcare costs all impact the three parties’ strategic choices. The three stakeholders (i.e., doctor, patient, and platform) influence each other’s behavior. Therefore, the platforms should pay attention to doctor qualification and information protection, improve the platform function and patient evaluation mechanism, and set reasonable prices for online healthcare treatment. Simultaneously, the government should increase supervision, regulate the behavior of the platform, clarify the distribution of responsibility for online healthcare legal issues, and promote the healthy development of OHP. This has significant implications for preventing the spread of COVID-19.

## Data availability statement

The original contributions presented in the study are included in the article/supplementary material, further inquiries can be directed to the corresponding author.

## Ethics statement

Ethical review and approval was not required for the study on human participants in accordance with the local legislation and institutional requirements. Written informed consent from the patients/participants or patients/participants legal guardian/next of kin was not required to participate in this study in accordance with the national legislation and the institutional requirements.

## Author contributions

LZ: conceptualization, methodology, writing. DL: software. WL: data curation. ZX: conceptualization, software. All authors have read and agreed to the published version of the manuscript.

## Conflict of interest

The authors declare that the research was conducted in the absence of any commercial or financial relationships that could be construed as a potential conflict of interest.

## Publisher’s note

All claims expressed in this article are solely those of the authors and do not necessarily represent those of their affiliated organizations, or those of the publisher, the editors and the reviewers. Any product that may be evaluated in this article, or claim that may be made by its manufacturer, is not guaranteed or endorsed by the publisher.
